# A Scoping Review Protocol on Senior Tourists’ Accommodation Preferences

**DOI:** 10.12688/f1000research.169592.1

**Published:** 2025-09-02

**Authors:** Ninad Sudhir Chavan, Senthilkumaran Piramanayagam, Naresh Nayak Perampalli, Harini KN, Anand P Ambali

**Affiliations:** 1 Welcomgroup Graduate School of Hotel Administration, Manipal Academy of Higher Education, Manipal, Karnataka, India; 2 Le Cordon Bleu, Te Aro, Wellington, 6011, New Zealand; 3 TA Pai Management Institute, Bengaluru, Manipal Academy of Higher Education, Manipal, Karnataka, India; 4 Shri B M Patil Medical College Hospital and Research Centre, BLDE Deemed to be University, Vijayapura, Karnataka, India

**Keywords:** Accommodation Preferences, Senior Tourism, Senior Tourists, Travel Preferences

## Abstract

**Background:**

The global demographic shift to an ageing population has placed individuals aged 60 and above as a crucial tourism market segment due to several factors. The senior tourism market, a growing segment, has distinct preferences and needs. Motivation for tourism, tourism-related needs, concerns for accessibility, travel companions, and past experiences determine the preferences of senior tourists. Comprehending these preferences is vital for the tourism industry to cater to this market segment effectively and sustainably. While researchers have extensively examined preferences, there is a lack of research on senior tourists’ accommodation preferences. This scoping review aims to map research trends and provide a future research scope on senior tourists’ accommodation preferences.

**Methods:**

The updated Joanna Briggs Institute (JBI) methodological guidelines were adopted for this scoping review protocol. The proposed scoping review will be aligned with the methodological framework proposed by Arksey and O’Malley, the Population, Concept, Context (PCC) framework, to develop the research questions, and the Theory, Context, Characteristics, and Methodology (TCCM) framework for synthesising, summarising and reporting. Studies indexed in Scopus and Web of Science (WoS) databases published in English from their origin to 2025, irrespective of study location are included. The articles will be screened independently by two reviewers for eligibility. The extracted data will be presented graphically and descriptively.

**Discussion:**

This scoping review maps the existing literature on senior tourists’ accommodation choices by adopting the TCCM framework. This study will provide future research directions associated with senior tourists’ accommodation preferences.

**Registration:**

This scoping review protocol was registered with the Open Science Framework (OSF). DOI: https://doi.org/10.17605/OSF.IO/B64TM

**Ethics and Dissemination:**

There is no direct involvement of human participants in this study on secondary data; therefore, ethical clearance is not required. The results of this review will be disseminated through journal publications and conference presentations in the future.

## Introduction

A significant shift towards an older population has been observed in the global demographics. The World Health Organization (WHO) highlights that, globally, the older population (aged 60 years and above) is projected to double—reaching approximately 2.1 billion by 2050—compared to one billion in 2020. The current growth in the older population is much faster than in the past. ^1^ Considering the present growth rate, the United Nations (UN) estimates that the population aged 65 years and above will surpass the population under 18 in the 2070s. ^2^ However, ageing comes with an increased risk of geriatric syndromes, malnutrition, disability, and poor health, resulting in a reduced quality of life and placing an enormous medical and healthcare burden on society. Beyond health-related risks, the elderly population faces ageism—defined as “ *the stereotypes (how we think), prejudice (how we feel), and discrimination (how we act) directed towards people on the basis of their age*”. ^3^ Ageism, which is ubiquitous worldwide, raises challenges of age-related discrimination, violence, stigma, abuse, isolation, and exclusion. Such circumstances prevent older adults from fully participating in society and exercising their basic human rights, thereby deteriorating their health and well-being. ^3^

To address the range of negative consequences associated with demographic changes in the elderly population and foster the sustainable development of society, the UN announced 2021–2030 as the ‘ *UN Decade of Healthy Ageing’*, focusing on age-friendly environments, tackling ageism, and guaranteeing inclusive and sustainable growth. ^4^ This initiative aligns with the second guiding principle of the 2030 Agenda for Sustainable Development, ‘ *Leave No One Behind’,* which encourages inclusivity and equity among all age groups. ^5^ Travel is considered a potential avenue for healthy ageing, improved health, well-being, and quality of life. The coronavirus disease (COVID-19) pandemic has reinforced the value of travel in public health beyond its economic significance. Specifically, travel is regarded as a non-pharmacological intervention for health and well-being among people of all ages, genders, health statuses, family situations, and economic conditions. ^6, 7^ The travel industry can potentially drive shared prosperity by advancing policies, frameworks, and governance, as well as by utilising tourism income to achieve sustainable development goals. ^8^

As the world’s second-largest industry, tourism is responsible for promoting healthy ageing by actively engaging with the older population. ^3^ A Eurostat report on tourism trends and ageing indicates that senior tourism accounted for nearly one in four tourism nights in 2022, highlighting the high level of tourism participation among the elderly. ^3, 9^ However, the COVID-19 pandemic had a devastating impact on tourism, particularly senior tourism. ^5^ On a positive note, a recent report by the World Tourism Organization ^10^ estimates that the tourism sector has nearly returned to pre-pandemic levels of international arrivals, contributing approximately USD 3.4 trillion to the global gross domestic product (GDP). Globally, the senior travel market is valued at USD 1.72 trillion in 2024 and is expected to reach USD 2.62 trillion by 2030, underscoring the need for tourism stakeholders to explore the opportunities associated with this market. ^11^ Given the growing number of seniors, this segment is likely to become one of the most significant segments within tourism in the coming decades. ^12^

The literature clearly indicates a growing interest in ageing, tourism, and the physical, mental, and social well-being of the elderly population. ^13, 14^ While senior tourism is becoming an important driver of tourism economic growth, scholars have pointed out the limited academic focus on the topic. ^15, 16^ Among the many decisions involved in senior travel, the choice of accommodation is considered a key determinant of their experience and significantly influences satisfaction. However, there is a notable research gap in senior tourists’ accommodation preferences. ^17, 18^ Furthermore, although some reviews have been published on senior tourism, travel motivation, and the influence of ageing, there is a scarcity of scoping reviews on senior tourists’ accommodation preferences. ^13, 14, 19, 20^ Understanding senior tourists’ accommodation choice attributes is essential for meeting their unique needs and designing services that cater to this market segment. This will contribute to both economic and social development, particularly in relation to seniors. ^17^ As the senior tourism segment continues to evolve rapidly, a scoping review is needed to provide a synthesised map of existing evidence on accommodation attribute preferences, research trends, theories and methodologies adopted, research gaps, and future directions. This study aims to identify the literature on senior tourists’ accommodation preferences and explore the relevant theories, contexts, characteristics, and methods to present an organised overview of the research.

## Method

### Protocol

According to the Joanna Briggs Institute (JBI) article on methodology for scoping review protocols ^21^ and the five-stage framework by Arksey and O’Malley, ^22^ this study will be conducted with reference to the Preferred Reporting Items for Systematic Reviews and Meta-Analyses extensions for Scoping Reviews (PRISMA-ScR). ^23^ The Preferred Reporting Items for Systematic Reviews and Meta-Analyses extensions for protocol (PRISMA-P) checklist, consisting of 17 items, was referred to and registered on the open-access repository Figshare. ^24, 25^ This protocol was registered with the Open Science Framework (OSF) ^26^ to communicate with the research community about the proposed scoping review. A Theory, Context, Characteristics, Methodology (TCCM) framework will be adopted to summarise and report the synthesised data. ^27^

1. **Identifying the research question**

Following the Population, Concept, and Context (PCC) framework, research questions were developed ( Table 1) and addressed in the scoping review, ensuring broader coverage of the relevant literature.

1) What are the global trends in research on senior tourists’ accommodation preferences?
2) What is the current state of research on senior tourists’ accommodation choices in terms of theories used, context studied (geographical and cultural settings), characteristics, and methodologies employed?
3) What are the future research scopes for senior tourists’ accommodation preferences?
2. **Identifying relevant studies**

**Table 1.** PCC * framework for developing a research question.

**Population**	Senior tourists aged 60 years and above
**Concept**	Senior tourists towards accommodation preferences
**Context**	No restrictions on the location and timeline of the study

* PCC: Population, Concept, Context.

The selection of related studies will be done using the PCC mnemonic to establish the inclusion and exclusion criteria for the review.

### Population

This review will consider studies related to senior tourists. Studies should include participants aged 60 years and above. The minimum age criterion of 60 years is set, as mentioned by the WHO. Studies comparing senior tourists with other age cohorts will also be included.

### Concept

Considering the increased population of older adults, the tourism industry considers senior tourists an important travel market segment. Research on the senior tourist segment is growing in the current scenario. During tours and travel, senior tourists demonstrate different motivations, preferences, technology adoption, and concerns for accessibility, health, and safety than other age cohorts. ^17, 20^ Thus, any research relating to seniors and focusing on the hospitality and tourism industry is of interest. Additionally, studies on the accommodation preferences of senior tourists will be included.

### Context

Studies published until August 2025 will be included in this review. To gain a comprehensive understanding of the trends in senior tourist research, this review purposefully considers studies published from its origin. There will be no limit on the geographical location considered in the study for inclusion in the review to maintain the broader scope of the review. Although the published research article domain should be among social sciences and humanities, it should not include other areas such as agriculture, medicine and engineering.

### Types of evidence sources

This study will include peer-reviewed articles which can be qualitative, quantitative, mixed-method, or interventional in nature to ensure the rigor of the research and its findings. However, it does not consider book chapters, conference papers and reports.

### Search strategy

Articles will be searched in two major databases, Scopus and Web of Science (WoS). The suitable terms mentioned in Table 2 for the search string of senior people, tourists, and the hospitality industry will be included, which were developed by consulting subject matter experts and professors. A broad study range was considered because no review has been conducted on senior tourists’ accommodation preferences to date. Search filters for language, document type, and research areas/domains will be applied.

3. **Study selection**

**Table 2.** Search terms.

Older people	Aged, older adults, elderly, seniors, older people, older tourists, geriatric, older population, aging population, senior tourists, senior citizens, older citizen
Tourists	Hotel guests, tourists, tourists, customers, consumers
Hospitality industry	Hospitality, travel industry, tourism, hotel industry, lodging, accommodation sector, hotel, lodging industry

Using the search string, the document list will be exported from both databases and uploaded to Rayyan software for deduplication and title-abstract screening. The screening will include two stages: title-abstract and full-text screening of each article, where two reviewers will assess the articles based on the specified criteria set according to the research objectives. Conflicts between reviewers will be resolved by discussing the article and review criteria together; a third reviewer will assist in cases of further ambiguity. A PRISMA flowchart will be created to elaborate on the number of articles searched, included, and excluded during the process. ^28^

4. **Charting the data**

The final included articles will be processed to retrieve information that will be compiled into a dataset. The data will be extracted and organised in Microsoft Excel to visualise details about the research problem, design, key findings, and implications. The format of the data extraction sheet is mentioned in Table 3, which will assist in reporting the data.

5. **Collating, summarising and reporting the results**

**Table 3.** Data extraction sheet.

Number	Item	Description
1	Title of the paper	What is the title of the article?
2	Journal	In which journal the article is published?
3	Year	When did the article got published?
4	Authors	Who all are the authors of the paper?
5	Problem addressed	What issue is focused upon to resolve?
6	Research design	What is the research method used?
7	Country of the study	Which country the study is performed in?
8	Age definition	What is the age criteria set in sampling/study?
9	Theory used	What existing theories are used/developed?
10	Findings	What are the major outcomes/results of the study?
11	Limitation	What are the limits/drawbacks of the study?
12	Future scope	What are future recommendations given in the study?
13	Theoretical implication	What theoretical benefits are obtained from study?
14	Managerial implication	What managerial/practical benefits are obtained from study?

Once the data extraction is complete, the collated data will be synthesised and summarised by visualisation and descriptive methods using the TCCM framework. Both quantitative and qualitative studies will be summarised descriptively and thematically.

We will focus on the first research question by identifying trends and themes of the selected articles and grouping them to understand existing research on senior tourists’ preferences. Additionally, to address further research questions and inform future recommendations, this study highlights unexplored gaps and key research areas, providing a foundation of existing knowledge and identifying opportunities for future studies on senior tourists’ accommodation preferences.

### Dissemination

We plan to disseminate the scoping review findings in peer-reviewed journals for publication and conference presentations.

### Study status

To date, we have completed the initial phase of conceptualising the idea and forming research questions, along with the search strategy and inclusion criteria. We formed a search string by running a pilot search in databases, and will proceed with the search, followed by deduplication and screening of the published articles.

## Discussion

A scoping review is the best approach for mapping and synthesising broader evidence and gaps across multiple disciplines. ^21^ To the best of our knowledge, no scoping review has been conducted in the field of senior tourism. Thus, we are conducting the first scoping review to portray the research trends and scope of senior tourists’ accommodation preferences. The proposed TCCM framework helps to structure the review systematically across four key dimensions: theories used, context studied, characteristics/variables examined, and methodologies adopted. We hope that this scoping review will provide insights into seniors’ accommodation preferences, considering their changing needs, and provide better services to the senior travel market segment. A thorough literature search will be conducted from its origin to the present to accurately trace research development and coverage in this area. This study will not consider the quality assessment of the articles, as it is not a mandatory criterion for conducting a scoping review; however, it will be counterbalanced by considering only peer-reviewed articles published in journals. This protocol provides a clear and transparent outline for conducting scoping reviews in this or any other relevant field. Articles published in English can be expected to be one of the limitations of this study; some studies may have important data that are available in different languages.

## Ethical considerations

This scoping review involves a study on secondary data, that is, existing published articles, and does not involve any human participants directly.

## Data Availability

There are no underlying data linked to this article. OSF: A Scoping Review Protocol on Senior Tourists’ Accommodation Preferences, https://doi.org/10.17605/OSF.IO/B64TM. ^26^ Figshare: ‘PRISMA-P’ checklist. DOI: https://doi.org/10.6084/m9.figshare.29898464. ^25^ Study data can be accessed according to the terms of the Creative Commons Attribution 4.0 International Licence (CC-BY 4.0).
