# Supplementary material for: A Scoping Review Protocol on Senior Tourists’ Accommodation Preferences
Source: F1000Res. 2025 Sep 2;14:852. [Version 1] doi: 10.12688/f1000research.169592.1 (PMC12930032; doi:10.12688/f1000research.169592.1)
Supplement: Peer review history

# Reviewer report: Reviewer response for version 1

**DOI:** 10.5256/f1000research.186946.r460047

**Published:** 2026-02-23

**Authors:** Guruprasad Vijayasarathi

**Affiliations:** 1 Associate Professor, Occupational Therapy Program, School of Rehabilitation and Medical Sciences, College of Health Sciences, university of nizwa, nizwa, Ad Dakhiliyah ‍Governorate, Oman

The review would benefit from a clearer operational definition of “senior tourists.” It is currently unclear whether this term refers to functionally independent older adults, those who use mobility aids (e.g., walkers or wheelchairs), or individuals with significant physical or cognitive impairments.

 Given that accommodation preferences may vary substantially according to functional status and type of health condition, greater clarity is essential. As this is a scoping review, the authors may consider explicitly limiting the population to healthy, community-dwelling, functionally independent older adults (chronic diseases permissible), while excluding individuals with established disabilities, whose tourism preferences may follow a different pattern and warrant separate analysis.

Is the study design appropriate for the research question?

Yes

Is the rationale for, and objectives of, the study clearly described?

Partly

Are sufficient details of the methods provided to allow replication by others?

Yes

Are the datasets clearly presented in a useable and accessible format?

Yes

Reviewer Expertise:

Older adults; Mobility and functional performance; Rehabilitation (neurological/geriatric); Independent living and community participation; Outdoor mobility and environmental accessibility; Falls prevention in older adults.

I confirm that I have read this submission and believe that I have an appropriate level of expertise to confirm that it is of an acceptable scientific standard.

# Reviewer report: Reviewer response for version 1

**DOI:** 10.5256/f1000research.186946.r416398

**Published:** 2025-10-17

**Authors:** Agnieszka Stanimir

**Affiliations:** 1 Wroclaw University of Economics and Business,, Wroclaw, Wroclaw, Poland

The study proposed by the authors, which consists of the scoping review, is significant in determining the determinants of the choice of accommodation places for senior tourists.

 As the authors indicated, the scoping review aims to identify research trends and future research scope on senior tourists' accommodation preferences. The justification of the presented purpose is done correctly and sufficiently. The design of the study is suitable for the three research questions posed:

 1) What are the global trends in research on senior tourists’ accommodation preferences?

 2) What is the current state of research on senior tourists’ accommodation choices in terms of theories used, context studied (geographical and cultural settings), characteristics, and methodologies employed?

 3) What are the future research scopes for senior tourists’ accommodation preferences?

 However, in this matter, I have a small suggestion for the authors to consider when determining the study population and then analysing the source texts concerning the perception of various forms of accommodation by seniors

 Seniors are an increasingly heterogeneous group every year due to their health conditions and the use of new technologies. If literature sources referring to seniors are sought during the study, it is necessary to break down the results (if possible) into at least two groups: people aged 60-69 and 70+. This is because younger seniors often used new technologies during their professional activity and had access to better health care than older seniors. The first indicated factor, i.e. new technologies, affects the ability to search for and compare accommodation places remotely and independently. On the other hand, the chance to benefit from better health care means that younger seniors are more likely to use accommodation services and will be more likely to use them in the future because they will be active as tourists.

 Therefore, what the authors write about in the introduction, i.e. "ageing comes with an increased risk of geriatric syndromes, malnutrition, disability, and poor health, resulting in a reduced quality of life and placing an enormous medical and healthcare burden on society" is perceived differently in the group of younger seniors and in the group of older seniors.

 For this reason, the authors' search for attributes of the choice of accommodation by senior tourists cannot be generalised to the entire generation group of people 60+. In a group with such an age range, there will be parents and their children; differences are always observed on this level.

 Both methods and datasets are appropriate for the study and the answers to the research questions.

Is the study design appropriate for the research question?

Yes

Is the rationale for, and objectives of, the study clearly described?

Yes

Are sufficient details of the methods provided to allow replication by others?

Yes

Are the datasets clearly presented in a useable and accessible format?

Yes

Reviewer Expertise:

multivariate data analysis, econometrics, social sciences,  analysis of behaviour and needs, tourism and the tourism sector

I confirm that I have read this submission and believe that I have an appropriate level of expertise to confirm that it is of an acceptable scientific standard.
